# A Comparison of Two Measures of HIV Diversity in Multi-Assay Algorithms for HIV Incidence Estimation

**DOI:** 10.1371/journal.pone.0101043

**Published:** 2014-06-26

**Authors:** Matthew M. Cousins, Jacob Konikoff, Devin Sabin, Leila Khaki, Andrew F. Longosz, Oliver Laeyendecker, Connie Celum, Susan P. Buchbinder, George R. Seage, Gregory D. Kirk, Richard D. Moore, Shruti H. Mehta, Joseph B. Margolick, Joelle Brown, Kenneth H. Mayer, Beryl A. Kobin, Darrell Wheeler, Jessica E. Justman, Sally L. Hodder, Thomas C. Quinn, Ron Brookmeyer, Susan H. Eshleman

**Affiliations:** 1 Department of Pathology, Johns Hopkins University School of Medicine, Baltimore, Maryland, United States of America; 2 Department of Biostatistics, School of Public Health, University of California Los Angeles, Los Angeles, California, United States of America; 3 National Institute of Allergy and Infectious Diseases, National Institutes of Health, Bethesda, Maryland, United States of America; 4 Department of Medicine, Johns Hopkins University School of Medicine, Baltimore, Maryland, United States of America; 5 Departments of Global Health and Medicine, University of Washington, Seattle, Washington, United States of America; 6 Bridge HIV, San Francisco Department of Health, San Francisco, California, United States of America; 7 Departments of Epidemiology and Medicine, University of California San Francisco, San Francisco, California, United States of America; 8 Department of Epidemiology, Harvard School of Public Health, Boston, Massachusetts, United States of America; 9 Department of Epidemiology, Johns Hopkins Bloomberg School of Public Health, Baltimore, Maryland, United States of America; 10 Department of Molecular Microbiology and Immunology, Johns Hopkins Bloomberg School of Public Health, Baltimore, Maryland, United States of America; 11 Department of Epidemiology, School of Public Health, University of California Los Angeles, Los Angeles, California, United States of America; 12 Department of Epidemiology and Biostatistics, University of California San Francisco, San Francisco, California, United States of America; 13 The Fenway Institute/Beth Israel Deaconess Medical Center/Harvard Medical School, Boston, Massachusetts, United States of America; 14 Laboratory of Infectious Disease Prevention, New York Blood Center, New York, New York, United States of America; 15 Graduate School of Social Work, Loyola University Chicago, Chicago, Illinois, United States of America; 16 Departments of Epidemiology and Medicine, Columbia University, New York, New York, United States of America; 17 Department of Medicine, Division of Infectious Diseases, New Jersey Medical School, Newark, New Jersey, United States of America; Asociacion Civil Impacta Salud y Educacion, Peru

## Abstract

**Background:**

Multi-assay algorithms (MAAs) can be used to estimate HIV incidence in cross-sectional surveys. We compared the performance of two MAAs that use HIV diversity as one of four biomarkers for analysis of HIV incidence.

**Methods:**

Both MAAs included two serologic assays (LAg-Avidity assay and BioRad-Avidity assay), HIV viral load, and an HIV diversity assay. HIV diversity was quantified using either a high resolution melting (HRM) diversity assay that does not require HIV sequencing (HRM score for a 239 base pair env region) or sequence ambiguity (the percentage of ambiguous bases in a 1,302 base pair pol region). Samples were classified as MAA positive (likely from individuals with recent HIV infection) if they met the criteria for all of the assays in the MAA. The following performance characteristics were assessed: (1) the proportion of samples classified as MAA positive as a function of duration of infection, (2) the mean window period, (3) the shadow (the time period before sample collection that is being assessed by the MAA), and (4) the accuracy of cross-sectional incidence estimates for three cohort studies.

**Results:**

The proportion of samples classified as MAA positive as a function of duration of infection was nearly identical for the two MAAs. The mean window period was 141 days for the HRM-based MAA and 131 days for the sequence ambiguity-based MAA. The shadows for both MAAs were <1 year. Both MAAs provided cross-sectional HIV incidence estimates that were very similar to longitudinal incidence estimates based on HIV seroconversion.

**Conclusions:**

MAAs that include the LAg-Avidity assay, the BioRad-Avidity assay, HIV viral load, and HIV diversity can provide accurate HIV incidence estimates. Sequence ambiguity measures obtained using a commercially-available HIV genotyping system can be used as an alternative to HRM scores in MAAs for cross-sectional HIV incidence estimation.

## Introduction

HIV incidence is the rate of new HIV infections in a population. Reliable incidence estimates are needed to monitor and respond to the HIV/AIDS epidemic. Longitudinal cohort studies and cross-sectional surveys have been used to estimate HIV incidence. Cross-sectional incidence estimation may be preferred in some settings [1].

Serologic assays have been developed for cross-sectional HIV incidence estimation. However, these assays can overestimate incidence because some individuals with long-term HIV infection are misclassified as assay positive [2]. Some investigators have suggested using sequence-based measures of HIV diversity for HIV incidence estimation (e.g., by quantifying the proportion of ambiguous or mixed base positions in Sanger sequencing data or by using computational methods to quantify HIV diversity using next generation sequencing data) [3], [4], [5], [6]. This approach is based on the premise that HIV diversity tends to increase over time following HIV infection [7], [8]. Potential limitations of using sequence-based diversity data alone for HIV incidence estimation have been noted [5]. The cost of this approach would also be prohibitive for large cross-sectional surveys.

Multi-assay algorithms (MAAs) have recently been developed that provide accurate cross-sectional HIV incidence estimates for populations in the United States (US), where most HIV infections are subtype B [9]. These MAAs include both serologic assays and non-serologic biomarkers, such as CD4 cell count and HIV viral load [2], [9], [10]. We recently developed a robust MAA that includes the BED capture enzyme immunoassay (BED-CEIA, Calypte Biomedical Corporation, Lake Oswego, OR, USA [11]), an avidity assay based on the Genetic Systems 1/2+O EIA (BioRad-Avidity assay; BioRad Laboratories, Redmond, WA, USA, [12]), HIV viral load, and HIV diversity [13]. An advantage of this MAA is that it does not require CD4 cell enumeration at the time of sample collection [13]. In this MAA, HIV diversity in the *env* region is quantified using a high resolution melting (HRM) diversity assay that does not require sequencing [14], [15]. The assay is less expensive and easier to perform than sequencing assays and simplifies data analysis, since the output of the HRM diversity assay is a single numeric HRM score. The HRM diversity assay has been validated by comparison of HRM scores to diversity measures obtained from next generation sequencing data [15]. In previous reports, this assay has been used to compare HIV diversity in individuals with recent vs. non-recent infection [8] and to analyze HIV diversification over time [15], [16]. The assay has also been used in studies that demonstrate the biological relevance of HRM-derived measures, including the association of HRM scores with infant survival [17] and response to antiretroviral treatment [18].

While the HRM diversity assay offers many advantages for measuring HIV diversity, it is not widely available. For this reason, we also evaluated the performance of a MAA that includes sequence ambiguity in the *pol* region as a measure of HIV diversity. Previous studies have used *pol* region sequence ambiguity to measure HIV diversity for HIV incidence analysis [3], [5], [19]. In this report, sequence ambiguity was quantified by measuring the percentage of ambiguous bases in *pol* region consensus sequences generated using an HIV genotyping system developed for HIV drug resistance testing (ViroSeq HIV-1 Genotyping System, Celera, Alameda, CA, USA). While this approach is more labor-intensive and costly than measuring HIV diversity using the HRM diversity assay, the ViroSeq system is commercially available and is used in a large number of laboratories in the US and elsewhere.

The two MAAs evaluated in this report include a limiting antigen avidity assay recently developed by the US Centers for Disease Control for HIV incidence estimation (LAg-Avidity assay, Sedia Biosciences Corporation, Portland, OR, USA [20]) rather than the BED-CEIA. The LAg-Avidity assay is combined with a second serologic assay, the BioRad-Avidity assay (described above), as well as two non-serologic biomarkers: HIV viral load and HIV diversity (HRM score for a region in HIV *env* or the level of sequence ambiguity in *pol* region data from population sequencing). Samples were considered to be MAA positive (likely from individuals with recent HIV infection) if they met the criteria for all of the assays in the MAA.

The performance of the two MAAs was assessed using a large set of samples from individuals in three clinical cohorts with known duration of HIV infection. Performance was assessed by evaluating: (1) the proportion of samples classified as MAA positive as a function of duration of infection, (2) the mean window period (the mean duration of time that individuals were MAA positive), (3) the shadow (the time period prior to sample collection that is being assessed by the MAA [2], [21]), and (4) the accuracy of MAA-derived cross-sectional incidence estimates for three cohort studies. The performance of these two MAAs was also compared to the performance of an optimized 2-assay MAA that does not include a diversity measure.

## Methods

### Ethics Statement

The Multicenter AIDS Cohort Study (MACS), AIDS Linked to the IntraVenous Experience (ALIVE), HIV Network for Prevention Trials (HIVNET) 001/001.1, Johns Hopkins Hospital Clinical Cohort (JHHCC), HIV Prevention Trials Network (HPTN) 061, and HPTN 064 studies were conducted according to the ethical standards set forth by the institutional review boards of the participating institutions and the Helsinki Declaration of the World Medical Association; participants provided written informed consent. The work reported here included analysis of stored samples and data from those studies; this work was approved by Institutional Review Boards at the participating institutions. No participants were recruited or followed during the course of this work.

### Samples used for MAA development

Stored plasma and serum samples collected 1 month to >8 years after seroconversion were acquired from cohort studies in the US (1,782 samples from 709 individuals, see Table S1). The sources of these samples were: the MACS [22] (men who have sex with men [MSM], 564 samples from 365 individuals), the ALIVE cohort [23] (persons who inject drugs, 410 samples from 241 individuals), and the HIVNET 001/001.1 vaccine preparedness cohort [24] (men and women with different risk factors for HIV acquisition, 808 samples from 103 individuals). Five hundred additional samples from the JHHCC that were collected >8 years after seroconversion were also analyzed [25]; approximately half of the JHHCC study participants are persons who inject drugs. Detailed descriptions of these sample sets and the methods used to estimate the seroconversion date for each sample were reported previously [9], [26].

### Samples used for cross-sectional incidence estimation

Stored plasma and serum samples used for cross-sectional incidence estimation were obtained from three cohort studies in the US: (1) the HPTN 064 cohort (low incidence) [27], (2) the HIVNET 001/001.1 cohort (medium incidence) [24], and (3) the HPTN 061 cohort (high incidence) [28]. These samples were collected at follow-up visits 6–18 months after study enrollment (see Table S1).

### Laboratory methods

Testing with the LAg-Avidity and BioRad-Avidity assays was performed previously [9], [10]. LAg-Avidity assay results are reported as normalized optical density units (OD-n). BioRad-Avidity assay results are reported as avidity index (%). Samples that had a LAg-Avidity result <2.9 OD-n and a BioRad-Avidity avidity index result <85% (N = 213) were tested with an HIV viral load assay. One hundred nineteen of those samples analyzed had a viral load >400 copies/mL, and 113 (95.0%) of the 119 samples were available for evaluation with the HRM diversity assay and ViroSeq system (the remaining six samples were depleted in prior testing). HRM scores were obtained for 111 (98.2%) of the 113 samples (1 from the MACS cohort, 7 from the ALIVE cohort, and 103 from the HIVNET 001/001.1 cohort; 2 samples failed analysis). Sequence ambiguity measures were obtained for 108 (95.6%) of the 113 samples (1 from the MACS cohort, 7 from the ALIVE cohort, and 100 from the HIVNET 001/001.1 cohort; 5 samples failed analysis).

The HRM diversity assay was performed as previously described [8], [14]. Briefly, a region of HIV *env* was amplified. A smaller *env* region (ENV1, 239 base pairs) was then amplified in a nested polymerase chain reaction (PCR) that included a fluorescent, duplex-dependent DNA dye (LCGreen Plus, BioFire Diagnostics, Inc., Salt Lake City, UT, USA). After the nested PCR step, the samples were analyzed using a LightScanner instrument (BioFire Diagnostics, Inc.); in this step, samples were warmed, and the fluorescent dye was released as the DNA duplexes melted. The negative derivative of fluorescence vs. temperature (–dF/dT) was plotted against temperature to yield the melting peak for each sample. The width of the melting peak (which corresponds to the level of genetic diversity in the amplicon) was reported as the ENV1 HRM score. HRM scores were determined using the DivMelt software package (DivMelt, ENV1 protocol) [29].

The ViroSeq HIV-1 Genotyping System was used to generate HIV *pol* sequence data using 6–7 primers and to manually edit assembled sequences to yield a single consensus sequence (1,302 base pairs). Mixed base positions were identified according to the manufacturer’s instructions. The final consensus sequence was exported in FASTA format. A Perl script was used to calculate the number of mixed base positions in each sequence and to determine the percentage of bases in each sequence that were ambiguous: sequence ambiguity (%) = [(number of mixed base positions)×(100)]/(total number of positions). FASTA sequence data were submitted to GenBank (National Center for Biotechnology Information, U.S. National Library of Medicine, Bethesda, MD, USA) and were assigned accession numbers KF729799-KF729936.

### Statistical methods

Samples were classified as MAA positive if they met the criteria for all component assays. Samples were classified as MAA negative if they failed to meet the criteria for one or more of the component assays. MAAs were evaluated using statistical methods described previously [2], [26]. For each MAA, the mean window period and shadow were calculated by fitting cubic splines to the data; confidence intervals were determined using blocked bootstrapping. The results were used to generate probability curves that show the proportion of MAA positive samples as a function of time since HIV seroconversion. The mean window period corresponds to the area under the probability curve [26]. As noted above, the shadow measures the time period prior to sample collection that is being assessed by the MAA. One can also think of the shadow as follows: among persons who are MAA positive (in the window period) at the time of the survey, the shadow represents the average duration of time that those persons already spent in the window period prior to the survey. Additional information about the methods used to calculate the mean window period and shadow is provided in a previous report [26].

Samples that were missing HRM diversity assay or sequence ambiguity results (sample not available or assay failure) were excluded from the analysis. The potential impact of these missing values was assessed using a secondary analysis that incorporated the partial information available for these samples (i.e., data from the serologic and viral load assays); this analysis assumed that samples missing diversity data and samples with diversity data were not systematically different with regard to the relationship between being MAA positive and the duration of infection.

### Incidence estimation

Incidence estimates for the HPTN 064, HIVNET 001/001.1, and HPTN 061 cohorts were calculated using the following formula: Incidence = [(# MAA positive samples)×(100)]/[(number uninfected individuals)×(mean window period)] [13]. Confidence intervals were calculated as previously described [13], [30]. Incidence estimates were evaluated by calculating the percent difference between the incidence estimate obtained using a MAA and the incidence estimate obtained from longitudinal cohort follow-up, where % difference = [(the absolute value of the MAA-based incidence estimate minus the longitudinal incidence estimate)×(100)]/(the longitudinal incidence estimate). Statistical analyses were performed using the R statistical programming language [31] or Mathematica (Wolfram Research, Champaign, IL, USA).

## Results

**Figure 1:**
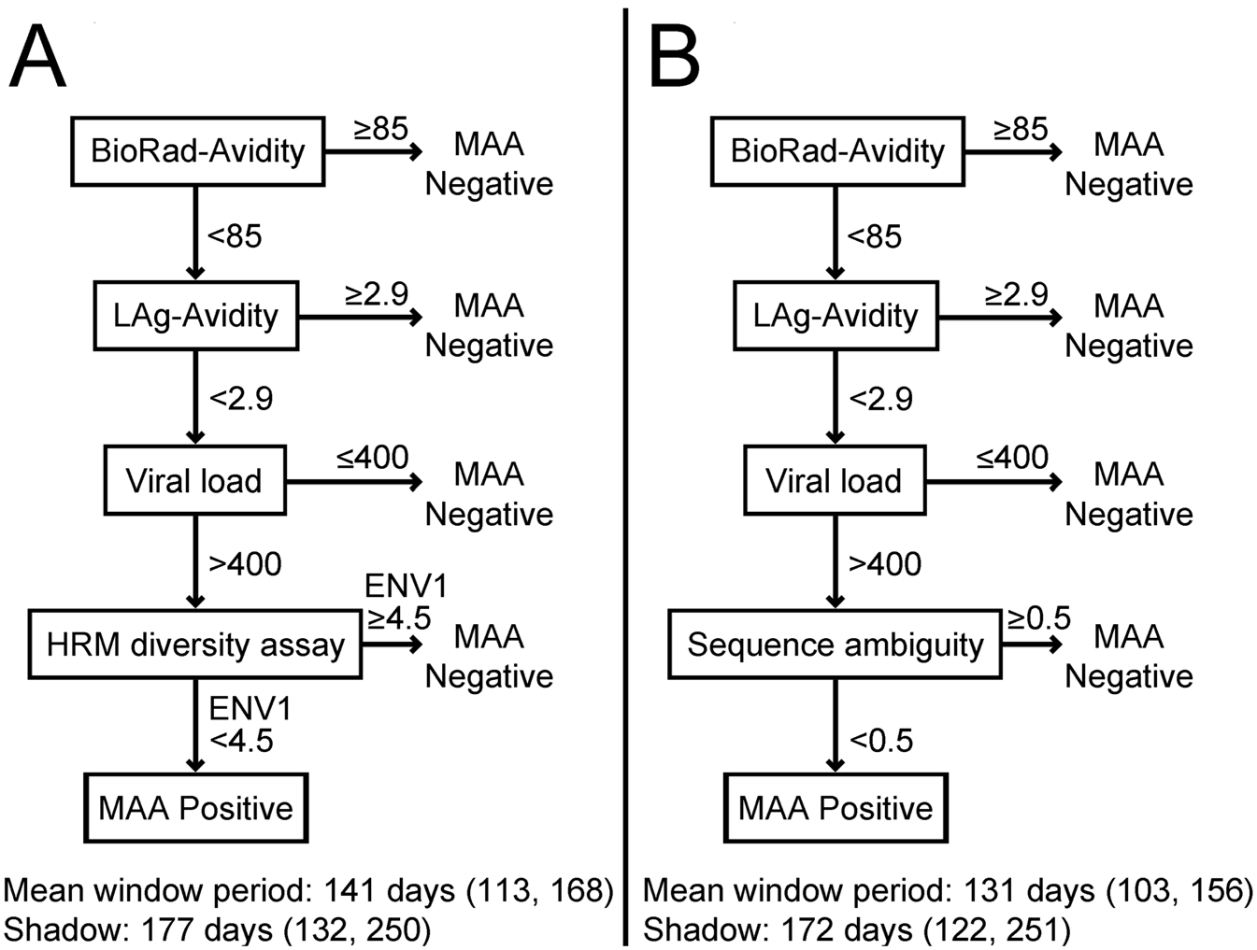
Multi-assay algorithms (MAAs) for cross-sectional HIV incidence estimation. Two MAAs are shown. The mean window period and shadow for each MAA are shown; 95% confidence intervals are shown in parentheses. Results from the component assays were expressed as follows: BioRad-Avidity assay: percentage (avidity index); limiting antigen avidity enzyme immunoassay (LAg-Avidity): normalized optical density units (OD-n); viral load: copies/mL; high resolution melting (HRM) diversity assay: single number (HRM score); sequence ambiguity: percentage.

We evaluated two new 4-assay MAAs that include the BioRad-Avidity assay, the LAg-Avidity assay, HIV viral load, and HIV diversity (measured using the HRM diversity assay or sequence ambiguity, Figure 1). Both MAAs used the following assay cutoffs: BioRad-Avidity assay <85%, LAg-Avidity assay <2.9 OD-n, and HIV viral load >400 copies/mL. The cutoffs for these three assays are the same as those in an optimized 4-assay MAA that also includes CD4 cell count [10]; that MAA was identified by comparing >500,000 candidate MAAs that included different assays and assay cutoffs [10]. The two new MAAs described in this report replace CD4 cell count in the optimized MAA with a diversity measure. One of the two new MAAs described in this report includes the HRM diversity assay for the ENV1 region as the fourth assay, using an HRM score cutoff value of <4.5 (Figure 1, Panel A). This HRM region and cutoff value were identified in a previous optimization study that evaluated MAAs that included the HRM diversity assay, the BED-CEIA, the BioRad-Avidity assay, and viral load [13]. The second new MAA described in this report includes sequence ambiguity analysis as the fourth assay, using an ambiguity cutoff value of <0.5% (Figure 1, Panel B). This cutoff value was used in a previous report that evaluated the use of sequence ambiguity alone for identification of recent HIV infections [3].

The two MAAs were evaluated using 1,782 samples from 709 individuals who enrolled in three cohort studies (MACS, ALIVE, and HIVNET 001/001.1; see Methods). The mean window period and shadow obtained for each MAA are shown in Figure 1. The MAA that included the HRM diversity assay had a mean window period of 141 days (95% CI: 113–168 days). The MAA that included sequence ambiguity had a mean window period of 131 days (95% CI: 103–156 days). Additional statistical evaluation of the two MAAs indicated that missing HRM data (for 8 samples) and missing sequence ambiguity data (for 11 samples) did not have a significant impact on the mean window periods determined for the MAAs (data not shown). These analyses did suggest that results generated using the MAA that includes the HRM diversity assay are more stable in the presence of missing data (data not shown). In a secondary analysis that accounted for missing data, the upper boundary of the 95% confidence interval for the shadow of the sequence ambiguity-based MAA was 456 days, indicating that this MAA may be evaluating incidence in a time period that extends more than 1 year before sample collection.

**Figure 2:**
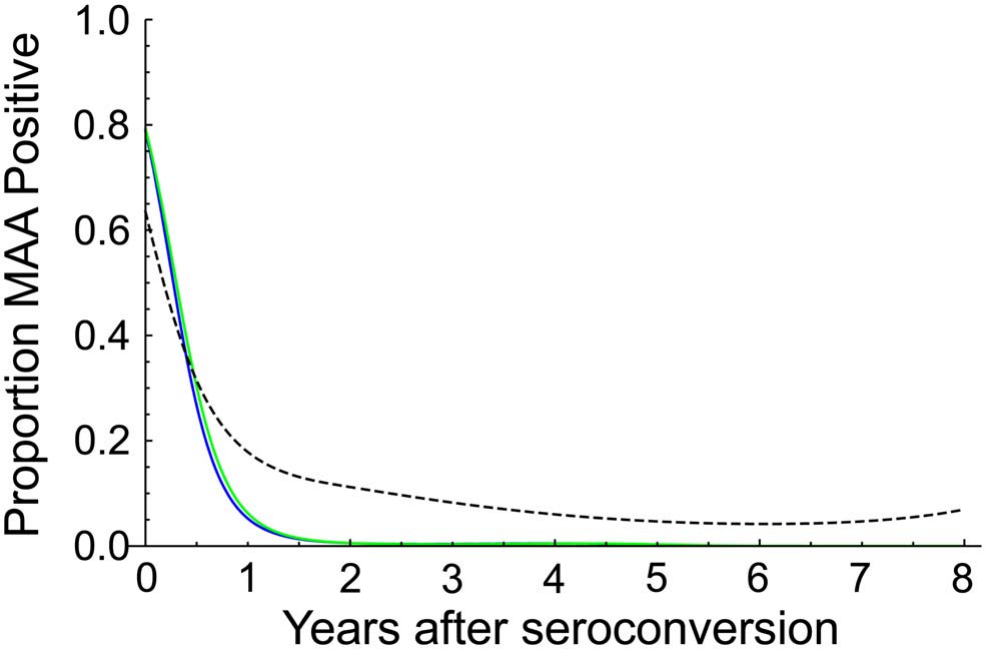
Proportion of samples classified as MAA positive as a function of the duration of HIV infection. Probability curves are shown for the two MAAs described in Figure 1. A probability curve is also shown for the limiting antigen avidity assay (LAg-Avidity assay cutoff<1.5 OD-n) alone [10]. Key: blue line, MAA with the high resolution melting (HRM) diversity assay; green line, MAA with sequence ambiguity; dotted line, LAg-Avidity assay alone.

Models for the probability of MAA positive classification as a function of duration of infection were generated for each of the two MAAs (Figure 2). For comparison, the figure also includes a model for the probability of assay positive classification using the LAg-Avidity assay alone (cutoff<1.5 OD-n) [10]. For both MAAs, the probability that samples were classified as MAA positive approached zero with increasing duration of infection (i.e., all individuals were eventually classified as MAA negative). This was not the case when the LAg-Avidity assay was used alone. For the MAAs, none of the 500 samples from individuals who were infected more than 8 years (samples from the JHHCC) were classified as MAA positive. In contrast, when the LAg-Avidity assay was used alone, 29 [5.8%] of the 500 samples were misclassified as assay positive [9], [10].

The two MAAs were also used to estimate HIV incidence in three clinical cohorts (HPTN 064, HIVNET 001/001.1, and HPTN 064; see Methods, Table 1). The number of samples tested in each step of the MAA, and the number of samples that met the criteria for each assay, are shown in Table 1. The number of serologic assays required for this type of assessment is influenced by the assay order. If the BioRad-Avidity assay is performed first (as shown in Figure 1 and Table 1), 301 (84%) of the 358 cohort samples are identified as MAA negative in the first step of the MAA, leaving 57 samples to be tested with the LAg-Avidity assay. Overall, 415 serologic assays are required. In contrast, if the LAg-Avidity assay is used as the first step in the MAA, only 270 (75%) of the 358 cohort samples are identified as MAA negative, leaving 88 samples to be tested with the BioRad-Avidity assay. Overall, 446 serologic assays would be required. The MAAs are also designed so that the more labor intensive and costly assays are performed last. For HPTN 064, HIVNET 001/001.1, and HPTN 061, diversity assays were only required for 2, 16, and 13 samples, respectively. For HIVNET 001/001.1, 1 of 16 samples was identified as MAA negative by sequence ambiguity. For HPTN 061, 4 of 13 samples were identified as MAA negative by the HRM diversity assay, and 3 of those 4 samples were identified as MAA negative with sequence ambiguity (one sample failed sequence analysis).

**Table 1:** Sample sizes used in calculating HIV incidence estimates for three clinical cohorts in the United States with two 4-assay MAAs.

		HPTN 064	HIVNET 001	HPTN 061
Length of follow-up (months)^a^		6 or 12^b^	18	12
# HIV negative		1,947	4,175	872
# HIV positive^c^		33	79^d^	246
Assays/Test results				
1. BioRad-Avidity assay	# evaluated	33	79	246
	# <85%	3	24	30
2. LAg-Avidity assay	# evaluated	3	24	30
	# <2.9 OD-n	3	20	24
3. Viral load	# evaluated	3	20	24
	# >400 copies/mL	2	16	13
4. HRM diversity assay	# evaluated	2	16	13
	# <4.5 (# MAA positive)	**2**	**16** e	**9** f
4. Sequence ambiguity	# evaluated	2	16	12^g^
	# <0.5 (# MAA positive)	**2**	**15** e	**9** f

Abbreviations: HPTN: HIV Prevention Trials Network; HIVNET: HIV Network for Prevention Trials; MAA: multi-assay algorithm; LAg-Avidity: limited antigen avidity assay; BioRad-Avidity: avidity assay based on the BioRad 1/2+O EIA; HRM: high resolution melting.

a Cross-sectional HIV incidence estimates were obtained by testing samples collected at the end of follow-up in three clinical cohorts: HPTN 064, HIVNET 001, and HPTN 061. The number of HIV-infected vs. HIV-uninfected individuals included in the cross-sectional survey is shown.

b Participants in HPTN 064 were followed for either 6 or 12 months.

c For HPTN 064, 33 study participants had samples available for analysis; 28 were seropositive at enrollment, one had acute HIV infection at enrollment, and four acquired HIV infection during the study. For HIVNET 001, 79 of 90 HIV-infected study participants had samples available for analysis; all 79 participants were HIV-uninfected at study enrollment. For HPTN 061, 246 participants had samples available for analysis; 218 were seropositive at study enrollment, three had acute HIV infection at enrollment, and 25 acquired HIV infection during the study.

d 73 of these 79 samples were among the 808 samples from HIVNET 001 that were used to determine the window periods and shadows for the MAAs (see Figures 1 and 2).

e One specimen classed as MAA positive by the HRM-based MAA was classified as MAA negative by the ambiguity-based MAA.

f One specimen that was classified as MAA negative by the HRM-based MAA was classified as MAA positive by the ambiguity-based MAA.

g One specimen failed analysis with sequence ambiguity. Because the MAA could not be completed, this specimen was excluded from incidence calculations.

The cross-sectional incidence estimates obtained using the two new MAAs are shown in Table 2. These estimates were nearly identical to incidence estimates based on longitudinal cohort follow-up (Table 2) [28], [32], [33]. For each cohort, the point estimates of incidence obtained with the MAAs were within the 95% confidence intervals of the corresponding longitudinal incidence estimates, further supporting the accuracy of the new MAAs. All six of the MAA-derived incidence estimates differed by <21% from the corresponding longitudinal incidence estimates (percent difference, Table 2).

**Table 2:** Performance characteristics of MAAs and comparison of cross-sectional incidence estimates to longitudinal incidence estimates obtained for three clinical cohorts.

	Longitudinal cohort	HRM-based MAA	Sequence ambiguity-based MAA	2-assay MAA (no diversity measure)*
Method description	Gold standard^a^	This report	This report	Previous report
Mean window period	–	141 (113, 168)	131 (103, 156)	119 (94, 144)
Shadow	–	177 (132, 250)	172 (122, 251)	247 (160, 339)
Incidence estimate				
HPTN 064	**0.24%** (0.07, 0.62)	**0.27%** (0.03, 0.98)	**0.29%** (0.03, 1.07)	**0.32%** (0.04, 1.17)
HIVNET 001	**1.04%** (0.70, 1.55)	**1.13%** (0.63, 1.93)	**1.14%** (0.62, 1.98)	**0.92%** (0.45, 1.73)
HPTN 061	**3.02%** (2.01, 4.37)	**2.67%** (1.20, 5.28)	**2.88%** (1.29, 5.70)	**4.57%** (2.37, 8.24)
Percent difference^b^				
HPTN 064	–	12.50%	20.83%	33.33%
HIVNET 001	–	8.65%	9.62%	11.54%
HPTN 061	–	11.59%	4.64%	51.32%
Relative survey size^c^		0.84	0.91	1.00 (Reference)

*Includes only LAg-Avidity and BioRad avidity assays; addition of viral load did not impact MAA performance.

Abbreviations: HRM: high resolution melting; MAA: multi-assay algorithm; HPTN: HIV Prevention Trials Network; HIVNET: HIV Network for Prevention Trials.

The table compares performance characteristics of the HRM-based MAA (Figure 1), the sequence-ambiguity-based MAA (Figure 1), and a 2-assay MAA described in a previous report [10]. The 2-assay MAA includes the LAg-Avidity assay (cutoff<2.8 OD-n) and the BioRad-Avidity assay (cutoff<40%); addition of HIV viral load to this MAA did not improve assay performance [10]. For each MAA, the table shows the mean window period, the shadow, and the cross-sectional incidence estimates obtained for each cohort. Methods used to calculate cross-sectional incidence estimates and confidence intervals have been described previously [13]. For each incidence estimate, data presented include the point estimate of incidence (bolded) and the 95% confidence intervals for the incidence estimate (parentheses).

a Longitudinal incidence estimates were obtained previously for the three cohorts, where longitudinal HIV incidence = (number of seroconversion events)/(number of person-years of follow-up) [28], [32], [33]. For HPTN 064 (low incidence cohort), longitudinal incidence was assessed over 6–12 months of follow-up (1,639 person/years); four seroconverters were identified. For HIVNET 001 (medium incidence cohort), longitudinal incidence was assessed between the 12- and 18-month follow-up visits (2,304 person years); 24 seroconverters were identified. For HPTN 061 (high incidence cohort), longitudinal incidence was assessed over 12 months of follow-up (926 person years); 28 seroconverters were identified.

b The cross-sectional incidence estimates obtained for each MAA were compared to the longitudinal incidence estimates. The percent difference was calculated by the following equation: [(absolute value of the cross-sectional incidence estimate minus the longitudinal incidence estimate)×(100)]/(the longitudinal incidence estimate).

c The relative survey size shows the size of a cross-sectional survey that would be needed for each of the two new MAAs to obtain the same precision that would be achieved using the previously optimized 2-assay MAA. Because both numbers are <1, a smaller survey would be needed using either of the two new MAAs.

As a final step, we evaluated how inclusion of the diversity measure (HRM score or sequence ambiguity) impacted the performance of the MAAs. If the diversity measure was simply removed from the MAA (leaving a non-optimized 3-assay MAA with the same cutoffs for the other three assays), the proportion of samples classified as MAA positive still approached zero (all of the samples from individuals infected >8 years were classified as MAA negative). However, some samples from individuals with long-term infection (4–8 years) were classified as MAA positive (data not shown). This is reflected in the longer mean window period (175 days) and longer shadow (411 days) of the non-optimized 3-assay MAA compared to the two MAAs that include a diversity measure. The shadow for this MAA means that incidence is being assessed more than a year before sample collection, which does not meet our pre-specified requirements for MAA performance. We also compared the two new MAAs to an optimized 2-assay MAA includes the BioRad-Avidity assay (cutoff<40%) and the LAg-Avidity assay (cutoff<2.5 OD-n) [10]. The performance of this MAA was not significantly impacted by the addition of viral load [10]. As shown in Table 2, the 2-assay MAA has a shorter mean window period than the two new MAAs (only 119 days; 95% CI: 94, 144) and a longer shadow (247 days, 95% CI: 160, 339 days). The incidence estimates for the 2-assay MAA differed from the longitudinal incidence estimates slightly more than the two new MAAs. Furthermore, the shorter mean window period of the optimized 2-assay MAA means that surveys would require larger sample sizes to achieve the same level of precision in incidence estimates as those obtained using the two new MAAs (see Table 2, Relative survey size).

## Discussion

This report demonstrates that HIV diversity is a useful biomarker for cross-sectional HIV incidence estimation when combined with other assays in a MAA. The HIV incidence estimates generated for three clinical cohorts using the two MAAs described in this report were nearly identical to point estimates of HIV incidence based on longitudinal follow-up. An advantage of these MAAs is that they do not include CD4 cell count data, which may be difficult to obtain in cross-sectional surveys. Therefore, these MAAs allow the entire incidence assessment to be conducted using stored plasma or serum samples.

In these MAAs, a hierarchical approach is used for testing. Serologic assays, which are less costly and easier to perform, are performed first, followed by HIV viral load. HIV diversity assessments are required only for the small subset of samples with results that fall below the assay cutoffs for the two serologic assays and above the cutoff for HIV viral load. The cutoffs used for the serologic assays (optimized in a previous MAA [10]) are higher than the cutoffs recommended when the LAg-Avidity and BioRad-Avidity assays are used in a single-assay format for HIV incidence estimation. In those cases, assay cutoffs are selected to balance detection of incident infections with exclusion of long-term infections. In contrast, in the MAAs presented in this report, higher cutoffs are used for the serologic assays to maximize detection of incident infections. Specificity is achieved by using the two serologic assays in combination, by excluding samples with low viral load, and by excluding samples with high diversity. The order in which the two serologic assays are performed impacts the cost of incidence estimation using these MAAs. Because the BioRad-Avidity assay identifies a higher proportion of the test samples as MAA negative than the LAg-Avidity assay (using cutoffs of <85% and <2.9 OD-n, respectively), fewer serologic assays may be required when the BioRad-Avidity assay is performed first.

In the MAAs described in this report, samples with viral loads <400 copies/mL are classified as MAA negative. Viral suppression is associated with misclassification by the LAg-Avidity assay [34] but has not been associated with misclassification using the BioRad-Avidity assay [35]. Inclusion of viral load in the MAAs is also helpful, since samples with very low HIV RNA levels are not likely to be evaluable using the HRM diversity assay or sequence-based assays, which require a minimal level of HIV RNA for reverse transcription and PCR amplification (RT/PCR). In samples that are amplifiable, one should also consider that low viral load may impact diversity measures due to bottlenecking if very few HIV RNA copies are used for RT/PCR. A previous study demonstrated that the HRM diversity assay is only affected by viral load if the number of copies of HIV RNA used for HRM analysis is very low (e.g., <50 copies input HIV RNA, corresponding to a viral load of <500 copies/mL for the methods used in this report) [17]. Therefore, low viral load is not likely to impact incidence estimates obtained using the HRM-based MAA. HIV diversity can also be impacted by clinical and biologic factors. For example, genetic bottlenecking can occur *in vivo* in individuals with advanced HIV disease [7], [8], [16] and in individuals with prolonged exposure to a non-suppressive antiretroviral drug regimen [36]. Higher levels of HIV diversity may also be observed early in infection if the multiplicity of infection is high (e.g., in persons who inject drugs) [37] or in cases of dual subtype HIV infection [38]. These factors should be considered when HIV diversity is used to assess HIV incidence.

In this study, similar performance was observed for the MAA that includes the HRM diversity assay and the MAA that includes sequence ambiguity. The characteristics of these two laboratory methods are shown in Table S2. Several factors should be considered when choosing an HIV diversity assay for inclusion in a MAA. The HRM diversity assay is easier, faster, and less costly to perform than HIV sequencing. Software has been developed that automates calculation of single numeric HRM scores from melting curve data, reducing the effort and variability associated with manual HRM score calculation [29]. In contrast, the use of sequencing data to quantify HIV diversity requires more complex sample and data analysis protocols. The HRM diversity assay uses the LightScanner instrument, which provides high resolution melting curves with a high degree of temperature stability. While DNA melting curve data can be obtained using other instruments (e.g., those designed for real-time PCR), those instruments typically provide lower resolution data and greater temperature variability [39], and data from those instruments have not been evaluated in MAAs for incidence determination. An advantage of using sequence ambiguity to quantify HIV diversity is that many laboratories perform HIV genotyping for resistance testing. The HIV genotyping system used in this report (the ViroSeq HIV-1 Genotyping System) is commercially available and is used in many laboratories in the US and elsewhere. In addition to providing information on antiretroviral drug resistance, sequences generated using the ViroSeq system can be used for phylogenetic analysis of HIV in the MAA-positive samples.

Regardless of the method use to quantify viral diversity for HIV incidence estimation, it is important to note that the level of genetic diversity varies considerably in different regions of the HIV genome [40]. The sequence ambiguity-based MAA described in this report uses sequence ambiguity measures from a defined portion of the HIV *pol* region. This region was selected for convenience since *pol* data from this region are generated when the ViroSeq HIV-1 Genotyping System is used for HIV resistance testing. The region used for analysis with the HRM diversity assay (ENV1) was selected in a previous study that compared the performance of eight different regions for inclusion in MAAs for HIV incidence estimation [13]. Performance of MAAs that include sequence ambiguity is likely to be different than the MAA described in this report if a different region were analyzed (e.g., a different portion of the *pol* gene or another gene).

The sequence ambiguity measure used in the MAA in this report is based on the percentage of mixed base positions detected in a consensus sequence derived from population sequencing. Detection of mixed base positions is impacted by numerous factors, including the methods and platform used for sequence analysis [41], [42]. Detection of mixed bases is also impacted by the amount of HIV RNA used for analysis and the efficiency of the reverse transcription and amplification steps used to generate amplicons for sequencing. Furthermore, even when the same platform and methods are used to generate consensus sequences (e.g., the ViroSeq HIV-1 Genotyping System used in this report), the percentage of mixed base positions detected may be impacted by variation in manual sequence editing, which involves subjective interpretation of electropherogram data. Different users may employ different approaches for sequence editing, and results may vary from user to user [41]. Quality control measures are required to minimize variation in HIV sequence data analysis that could impact sequence ambiguity measures [43], [44]. Increased cost and labor and more complex data management protocols would be required if next generation sequencing were used to generate diversity measures for cross-sectional incidence studies. Regardless of the method used to obtain diversity measures for cross-sectional incidence analysis, strict quality control is needed to ensure the reproducibility of the data. Furthermore, if the methods used to quantify HIV diversity are different from those used in this report (e.g., different genomic region, different sequencing platform, or alternate sequencing approach) the assay or MAA would need to be validated using large sample sets, similar to the approach used in this report.

Both the HRM diversity assay and the sequence ambiguity assay use DNA primers for reverse transcription and PCR. The ViroSeq system also uses DNA primers for HIV sequencing. The HRM diversity assay has been used successfully for analysis of subtype A, B, C, and D HIV with relatively few assay failures [14], and the ViroSeq system performs well across a wide range of HIV subtypes [45]. In this report, which was based on analysis of subtype B HIV, very few samples failed analysis with either assay.

This study only included samples from the US, which are likely to be from individuals with subtype B infection. Further studies are needed to evaluate the performance of HIV diversity-based MAAs in populations infected with other HIV subtypes since serologic assays may perform differently in some subtypes [46], [47], [48]. Additionally, the majority of the samples in this study were from MSM. The viral populations in MSM may differ from those in individuals infected through heterosexual contact or injection drug use [37], [49], impacting HIV diversity measures. It is noteworthy that a previous study demonstrated that HRM scores were similar in a US cohort (subtype B, MSM) and a cohort from Malawi (subtype C, women), suggesting that HRM score results may not be substantially impacted by differences in mode of infection, subtype, or gender [16].

In summary, this report describes novel MAAs for cross-sectional HIV incidence estimation in US populations. Future studies will explore the use of HIV diversity-based MAAs for analysis of HIV incidence in populations with other HIV subtypes.

## Supporting Information

Table S1 **Source of samples used for analysis.**
(PDF)

Table S2 **Testing considerations for the HRM diversity assay and sequence ambiguity analysis.**
(PDF)
