# Supplementary material for: A Comparison of Two Measures of HIV Diversity in Multi-Assay Algorithms for HIV Incidence Estimation
Source: PLoS One. 2014 Jun 26;9(6):e101043. doi: 10.1371/journal.pone.0101043 (PMC4072769; doi:10.1371/journal.pone.0101043)
Supplement: Table S1 — Source of samples used for analysis. (PDF) [file pone.0101043.s001.pdf]

Table S1. Source of samples used for analysis.

Study	Reference	Population enrolled	HIV status at enrollment	Enrollment (years)	Follow-up interval	Length of follow-up	HIV test method
MACS	Kaslow (22)	MSM	Infected (42%)<br>Uninfected (58%)	1984-1985;<br>1987-1990;<br>2001-2003	6 months	Ongoing<br>(up to 29 years)	EIA / WB[a]
ALIVE	Vlahov (23)	PWID	Uninfected	1988-1989;<br>1994-1995;<br>1998; 2000;<br>2005-2008	6 months	Ongoing<br>(up to 25 years)	EIA / WB
JHHCC	Moore (25)	Various	Infected	1989-present	Various	Ongoing<br>(>24 years)	SOC
HIVNET 001	Celum (24)	Various	Uninfected	1995-1998	3 months	Up to 3 years	EIA / WB[b]
HPTN 061	Koblin (28)	Black MSM	Infected (25%)<br>Uninfected (75%)	2009-2010	6 months	12 months	Rapid test / WB[b]
HPTN 064	Hodder (27)	Women	Infected (1.5%)<br>Uninfected (98.5%)	2009-2010	6 months	6 or 12 months	Rapid test / WB[b]

Abbreviations: MACS: Multicenter AIDS Cohort; ALIVE: AIDS Linked to the Intravenous Experience; JHHCC: Johns Hopkins Hospital Clinical Cohort; HIVNET: HIV Network for Prevention Trials; HPTN: HIV Prevention Trials Network; MSM: men who have sex with men; PWID: persons who inject drugs; SOC: standard of care; EIA: enzyme immunoassay; WB: Western blot.

[a] Some participants were enrolled in this study before HIV testing algorithms were available. For these participants, stored samples from their baseline visit were tested when the tests became available.

[b] Retrospective quality control testing was performed at a centralized reference laboratory. This analysis included testing for acute infection at the visit prior to seroconversion.
