# Supplementary material for: A Comparison of Two Measures of HIV Diversity in Multi-Assay Algorithms for HIV Incidence Estimation
Source: PLoS One. 2014 Jun 26;9(6):e101043. doi: 10.1371/journal.pone.0101043 (PMC4072769; doi:10.1371/journal.pone.0101043)
Supplement: Table S2 — Testing considerations for the HRM diversity assay and sequence ambiguity analysis. (PDF) [file pone.0101043.s002.pdf]

Table S2. Testing considerations for the HRM diversity assay and sequence ambiguity analysis.

	**HRM diversity assay (ENV1 region)**	**Sequence ambiguity based on the ViroSeq HIV-1 Genotyping System**
Availability	Limited	Widely available
General use	Research use only	Research and clinical use (FDA-cleared for resistance testing)
Approximate cost/sample*	~$23	~$165
Typical batch size	46 samples	12 samples
Sample requirement	0.5 ml plasma (other sample types can be used)	0.5 ml plasma
Subtype considerations	Used successfully for analysis of HIV subtypes A, B, C, and D	Performs well across a wide range of HIV subtypes and variants
Region analyzed	239 bp *env* region (corresponds to codons for heptad repeat 1 [HR1] of gp41 and adjacent regions	1,302 bp *pol* region (corresponds to protease codons 1-99 and RT codons 1-335)
Testing schema and time required for analysis (estimate)	For 46 samples (total ~16 hrs):<br>Sample prep/extraction: 4 hrs; RT/PCR: 6 hrs; DNA purification: 4 hrs; nested PCR: 1.5 hrs; sample melting: 10 minutes; data extraction: 1 minute; data analysis: 5 minutes	For 12 samples (total ~32 hrs):<br>Sample prep/extraction: 2.5-3 hrs; RT: 2 hrs; PCR: 5 hrs; cycle sequencing: 5 hrs; automated sequence detection: 14-15 hrs; software analysis: 1-2 hrs (5-15 minutes/sample); calculation of sequence ambiguity from consensus sequences: 5 minutes
Reagents required	Commercially available (reagents for RNA extraction and melt curve analysis) plus home brew (reagents and primers for PCR amplification)	Commercially available
Specialized equipment required	Thermal cycler (generic) and LightScanner instrument (BioFire Diagnostics, Inc.)	Specific thermal cyclers and automated sequencers are approved for use
Raw data output	DNA melt curves	DNA sequences (from 6-7 overlapping primers) assembled to yield a single consensus sequence
Software required	DivMelt software package (DivMelt, ENV1 protocol); available free at CRAN (http://cran.r-project.org/web/packages/DivMelt/index.html)	Included with the ViroSeq system; Perl script used to calculate sequence ambiguity
Final data output	Single numeric HRM score	Sequence ambiguity (%)
Other information provided	--	ARV drug susceptibility (resistance report); consensus sequences can be used for phylogenetic analysis and HIV subtyping

Footnotes for Table S2

Abbreviations: HRM: high resolution melting; ENV: envelope; FDA: United States Food and Drug Administration; HR1: heptad repeat 1; bp: base pairs; RT: reverse transcription; PCR: polymerase chain reaction; hrs: hours; ARV: antiretroviral.

*Cost estimates include kits and reagents. These estimates do not include disposables such as gloves, tubes, and pipette tips.
